# Strategies to estimate the characteristics of 24-hour IOP curves of treated glaucoma patients during office hours

**DOI:** 10.1186/s12886-016-0191-7

**Published:** 2016-01-27

**Authors:** Leonardo Colombo, Paolo Fogagnolo, Giovanni Montesano, Stefano De Cillà, Nicola Orzalesi, Luca Rossetti

**Affiliations:** Eye Clinic, San Paolo Hospital, University of Milan, Via A. Di Rudinì 8, 20142 Milan, Italy

**Keywords:** Glaucoma, 24-hour IOP, Office-hour IOP, IOP peak and fluctuation, Timolol, Latanoprost, Brimonidine, Fixed combination timolol dorzolamide

## Abstract

**Background:**

It is known that office-hour measurements might not adequately estimate IOP mean, peaks and fluctuations in healthy subjects. The purpose of the present study is to verify whether office-hour measurements in patients in different body positions can estimate the characteristics of 24-hour intraocular pressure (IOP) in treated POAG patients.

**Methods:**

The 24-hour IOP curves of 70 eyes of 70 caucasian patients with treated glaucoma were analyzed. Measurements were taken at 9 AM; 12, 3, 6, and 9 PM; and 12, 3, and 6 AM, both in the supine (TonoPen XL) and sitting (Goldmann tonometer) positions. The ability of five strategies to estimate IOP mean, peak and fluctuation was evaluated. Each method was analyzed both with regression of the estimate error on the real value and with “hit or miss” analysis.

**Results:**

The least biased estimate of the Peak IOP was obtained using measurements from both supine and sitting positions, also yielding the highest rate of correct predictions (which was significantly different from 3 of the remaining 4 strategies proposed, *p* < 0.05). Strategies obtained from the combination of supine, sitting and peak measurements resulted to be least biased for the Mean IOP and the IOP Fluctuation estimate, but all strategies were not found significantly different in terms of correct prediction rate (the only significant difference being between the two strategies based on sitting or supine measurements only, with the former being the one with the highest correct prediction rate).

**Conclusions:**

The results of this study remark the concept that IOP is a dynamic parameter and that intensive measurement is helpful in determining its characteristics. All office-hour strategies showed a very poor performance of in correctly predicting the considered parameters within the thresholds used in this paper, all scoring a correct prediction rate below 52 %.

## Background

Intraocular pressure (IOP) is the main risk factor for the development and progression of glaucoma [1–4] and the only treatable one. IOP parameters (mean, peak and fluctuation) should be measured at diagnosis and strictly monitored in order to address the efficacy of IOP-lowering interventions. Mean IOP has been consistently recognized as a major risk factor for glaucoma and its progression [1, 2, 4] whereas the role of peak [5, 6] and fluctuation [7–12] as independent risk factors is still controversial.

Many studies report that mean IOP is not significantly different when measured during office-hour and 24-hour [13–16], but that office-hour data may significantly underestimate IOP peak and fluctuation: the majority of glaucoma patients had their IOP peaks outside office hours, most frequently occurring in night hours [14, 16–19].

In addiction to that supine office hour IOP measurements were described to better estimate IOP peaks than sitting measurements alone [16].

The most precise procedure to investigate IOP characteristics is 24-hour phasing [13–16, 20, 21] though it is unpractical, expensive and can be performed in a small subgroup of patients in few institutions. In addiction to that, due to the unavailability of IOP-measuring techniques that can be used while the patient sleeps, night-time evaluations require awakening of patients, potentially causing artifacts related to stress.

In 2009 the group of Leonardi developed a disposable contact lens sensor (CLS) that allows continous IOP monitoring (Sensimed AG, Lausanne, Switzerland) [22]. Nowadays the major limitation of this technology is the fact that the results of IOP evaluations are not provided in the habitual mmHg units but in an arbitrary unit and a direct comparison between the two methods cannot be performed yet [23].

The difficulty in obtaining 24-hour curves and the possible discrepancies between 24-hour and office-hour data led our [14] and other groups [16, 18, 19] to develop strategies to estimate 24-hour parameters by office-hour data. We showed that the collection of supine and sitting office-hour measurements may enhance the correct identification of 24-hour IOP characteristics in both healthy subjects and untreated POAG [14].

The purpose of the present study was to verify whether office-hour measurements taken in different body positions can estimate the characteristics of 24-hour IOP in POAG patients using different IOP-lowering treatments.

## Methods

This study was a retrospective analysis of 24-hour IOP curves of POAG treated patients, collected in the context of clinical trials investigating the circadian effect of antiglaucoma drugs. It was conducted at the Eye Clinic of San Paolo Hospital, University of Milan, Italy, after approval by the local Ethics Committee of San Paolo Hospital in Milan, and according to the tenets of the Declaration of Helsinki and national laws for the protection of personal data. Written informed consent was obtained from all the study participants.

### Study population

Seventy caucasian patients were enrolled (39 men and 31 women): 19 of them were treated with timolol (twice a day), 29 with latanoprost (once a day), ten with brimonidine (twice a day) and 12 with the fixed combination dorzolamide/timolol (FCDT, twice a day). These treatments options were part of their standard care.

To be included in the study, patients had to have glaucomatous visual fields (abnormal mean defect and corrected pattern standard deviation on at least two consecutive, reliable Humphrey 30–2 full-threshold tests), optic nerve head (ONH) changes (presence of concentric enlargement of the optic cup, localized notching, or both, as evaluated by means of color stereophotographs), and/or retinal nerve fiber layer (RNFL) defects (presence of focal or diffuse neuroretinal rim thinning, as evaluated by means of a scanning laser ophthalmoscope). Patients with ocular hypertension were excluded. Patients with untreated POAG were not included in this study.

Exclusion criteria included angle-closure glaucoma, secondary glaucomas, corneal abnormalities preventing reliable IOP measurement, previous filtration surgery, having one eye, pregnancy, significant disturbances of wake-sleep rhythms, and/or the regular use of hypnotic drugs reported by the patients. Eligibility was verified by means of a complete ophthalmic assessment.

### Twenty-four-Hour IOP evaluation

**Fig. 1:**
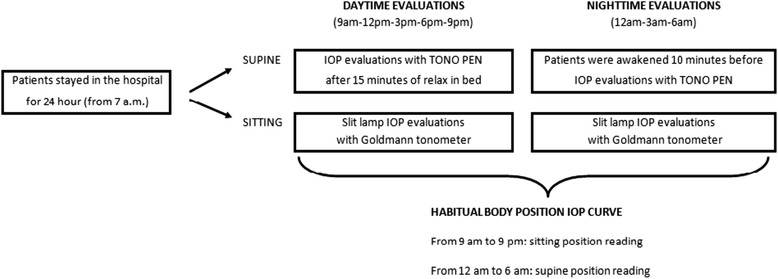
24-hour IOP evaluation

The methodology to assess 24-hour IOP is described in previous papers [14, 24] and summarized in Fig. 1.

The patients were hospitalized in the morning at 7 AM and stayed for the following 24 h. The awake period lasted from approximately 6:30 AM to 11:00 PM. IOP was measured at 9 AM; 12,3,6, and 9 PM; and 12, 3 and 6 AM both in the supine and sitting positions.

For the daytime measurements (9 AM–9 PM), patients were asked to go to bed and relax for approximately 15 min, after which supine IOP was measured in both eyes. After approximately 10 min, a second IOP value was measured at the slit lamp. During the night, the patients were awakened approximately 10 min before each measurement to prevent a sudden increase in IOP. The IOP supine measurements were taken with a handheld electronic tonometer (TonoPen XL; Bio-Rad, Glendale, CA); the IOP sitting measurements on the other hand were taken with Goldmann applanation tonometer at the slit lamp. Every measurement by TonoPen XL consisted of a variable number of readings until the coefficient of variation was less than 5 %. All measurements were taken at each time point by two well-trained glaucoma specialists who had obtained good accordance between their measurements (κ = 0.82 with both tonometers). If the measurements differed by >2 mm Hg, a third measurement was taken; the mean of two or the median of three recordings was used for the analysis.

### Peak IOP estimator strategies

Five parameters were tested in their ability of extrapolating peak IOP from office-hour readings:

1. The highest value obtained from the office-hour curve in the sitting position.
2. The formula proposed by Mosaed et al. 21 based on office-hour supine IOP (peak IOP = 5.98 + 0.771 + average office-hour supine IOP).
3. The formula proposed by Mosaed et al. 21 based on office-hour sitting IOP (peak IOP = 12.04 + 0.616 + average office-hour sitting IOP).
4. The mean of values obtained with the previous two formulas.
5. The highest value obtained from the office-hour curve in both supine and sitting positions.

### Mean IOP and IOP fluctuations estimator strategies

The 24-hour mean IOP and IOP fluctuations in habitual body position were compared to those calculated from:

1. Office-hour readings only in the sitting position (four measurements).
2. Office-hour readings only in the supine position (four measurements).
3. Office-hour sitting readings (four measurements) + the peak IOP, as estimated with the better of the previous formulas.
4. A combination of sitting and supine office-hour readings (four + four measurements).
5. A combination of sitting and supine office-hour readings (four + four measurements) + the estimated peak IOP.

### Statistical analysis

We considered the 24-hour curves obtained in habitual body position—that is sitting readings during waking time (from 9 AM to 9 PM) and supine readings during night time (from 12 to 6 AM). These curves were compared to the readings of the same 24-hour curves obtained during office hours (from 9 AM to 6 PM) in both supine and sitting positions in order to evaluate the ability of off-h readings to predict 24-h characteristics.

The following parameters were calculated: mean and range of the difference between estimate and 24-hour IOP parameter (expressed as absolute values, i.e., both an underestimation of −4 mm Hg and an overestimation of +4 mm Hg counting as 4 mm Hg. Linear models and generalized linear models were used to assess the quality of the analyzed estimators. First, we analyzed the relation between the real value to be estimated and the estimate error (specifically the difference between the estimate and the real value). For most of the cases, a linear regression approach properly modeled the relation between the real value and the estimate. For two strategies (Strategy 1 for the peak value and Strategy 1 for the fluctuations) a Zero Inflated Compound Poisson Model (ZICP) was used to model the estimate error dependency on the real value due its peculiar skewed distribution and high zero counts. A ZICP model can be broken down in two parts: the first part uses a binomial distribution to model the zero/non zero outcome of the Estimate Error, while the second part models the distribution of the continuous Estimate Error value when not zero.

Next, we adopted a “hit or miss” approach to assess the error rate of each strategy, considering an estimate error within ± 2 mmHg as a correct prediction for the Peak IOP and Mean IOP, ± 1 mmHg for IOP Fluctuations. In this context, a multinomial logit model was used on the various strategies to assess the odds of overestimation, underestimation and correct prediction. For the two strategies for which the ZICP was used, a completely correct prediction odds (0 mmHg error) was also derived from the binomial part of the model.

All numeric predictors (i.e. the real values) were mean centered, so the intercept corresponds to the estimates for the mean predictor value.

Then, an analysis by treatment groups was conducted to assess differences in estimation. In this case a classical ANOVA approach was chosen and a post-hoc correction (Tukey-Kramer) was used for multiple comparisons.

Finally, an overall comparison of the strategies was performed fitting a logit model for each feature considered (Mean, Peak and Fluctuation) with the Strategy as the predictor (where each strategy represented a level) and “Hit” or “Miss” as the response variable, according to the above classification of hits or misses. A Subject random effect was included to account for the fact that each strategy estimated the same real value for each subject. Then a post hoc analysis (Tukey-Kramer) was performed to compare the various strategies in terms of odds of hits or misses.

All calculations were made in R scripting environment.

## Results

The mean age was 73.1 ± 9.09 years. 24-hour IOP data in habitual body position are shown in Table 1.

**Table 1:** The 24-hour IOP data in Habitual Body Position

				Timing of peaks	
	Mean ± SD (Range)	Peak ± SD (Range)	Fluctuation ± SD (Range)	During office hours	Outside office hours
All patients	18.1 ± 3.4 (11.8–28.9)	22.5 ± 4.1 (15–33)	8.7 ± 2.9 (4–17)	35 %	65 %
Timolol	19.4 ± 3.5 (15.5–28.9)	24 ± 4 (19–33)	9 ± 2.7 (6–16)	42 %	58 %
Latanoprost	17.2 ± 3 (11.8–25.4)	21.7 ± 3.4 (16–29)	8.5 ± 2.7 (4–15)	24 %	76 %
Brimonidine	18.7 ± 3 (15.4–24)	22.5 ± 3,3 (19–28)	8.1 ± 2.2 (4–12)	40 %	60 %
DTFC	17.6 ± 3.9 (13.4–26.2)	22.2 ± 5.7 (15–32)	9 ± 4,3 (4–17)	42 %	58 %

Mean and peak IOP were lower in patients treated with latanoprost than in patients treated with timolol (respectively *P* = 0.03 and 0.05); significant differences were not found among the other groups. Differences in fluctuation between groups were negligible.

IOP peaked outside office hours in 65 % of all patients (timolol, 58 %; latanoprost, 76 %; brimonidine, 60 %; FCDT, 58 %).

The analysis focused on the accuracy of predictions from the various methods tested. Each method was analyzed both with regression of the estimate error (*estimated value – real value*) on the real value and with “hit or miss” analysis. For each of the three variables analyzed (Mean, Peak and Fluctuation) no significant differences were found among the treatment groups (*p*-values all greater than 0.13, ANOVA).

### Regression analysis

#### Peak estimate

The five strategies presented above were analyzed, in terms of difference of the estimated value from the corresponding real value (Estimate Error). Strategies from 2 to 5 were analyzed using simple regression with Gaussian error distribution using the real Peak IOP value as a predictor of the Estimate Error. The results are reported in Table 2. All strategies showed a significant dependency of the Estimate Error on the real Peak value with a negative slope, i.e. overestimation was more likely for smaller values and underestimation for larger values. Strategy 5 showed the smallest slope, yielding the least biased estimate of the Peak value.

**Table 2:** Estimated regression coefficients of the Estimate Error on the real IOP Peak value. In brackets, the standard errors of the coefficient estimates

	*Estimate Error*			
	Strategy 2	Strategy 3	Strategy 4	Strategy 5
IOP Peak Coefficient	−0.561***	−0.559***	−0.560***	−0.225**
	(0.056)	(0.045)	(0.045)	(0.089)
Global Mean	−1.858***	0.389**	−0.734***	0.229
	(0.228)	(0.181)	(0.182)	(0.361)
Observations	70	70	70	70
R^2^	0.594	0.696	0.696	0.086
Adjusted R^2^	0.588	0.691	0.692	0.072
Residual Std. Error (df = 68)	1.905	1.518	1.520	3.024
F Statistic (df = 1; 68)	99.566***	155.494***	155.859***	6.376**

Asterisks represent the significance level according to the legend in the footnotes

*Note:* *p < 0.1; **p < 0.05; ***p < 0.01

**Fig. 2:**
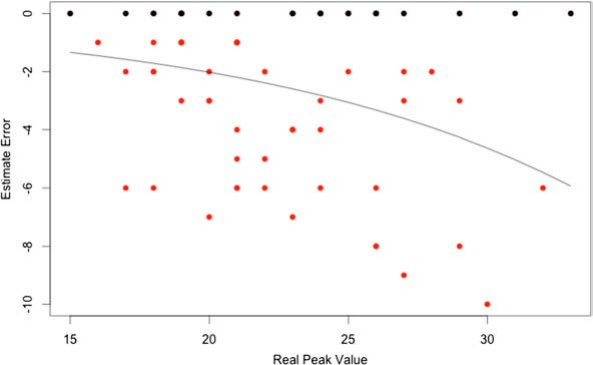
Scatterplot for Estimate Error of Strategy 1. *Black dots* represent the correct estimates (zero error), red dots the incorrect estimates. The *black line* represents the prediction from the ZICP model showing the non linear dependency of the non zero error on the real peak value. Note the skewed distribution around the mean

Strategy 1 required a detailed analysis due to its peculiar Estimate Error distribution: only underestimation errors were allowed with a relatively high rate of correct prediction (zero Estimate Error). To properly model such negatively skewed distribution with high zero counts we reversed the sign of the Error and used a ZICP model. This allowed accurate modeling of the odds of correct predictions and of the skewed negative errors. Particularly, the odds of correct prediction did not depend on the peak value (*p*-value = 0.3) and was significantly different from 1 (odds = 0.52, probability of correct prediction = 0.34, *p*-value < 0.01). The mean prediction for non zero errors was significantly dependent on the real peak IOP value (*p* < 0.001) in a non linear fashion, as depicted in Fig. 2.

#### Mean estimate

Five strategies for estimating the Mean IOP value were analyzed as for the Peak strategies. In this case only linear regression analysis was necessary. Results are presented in Table 3. Strategies 3 and 4 showed a non significant dependency on the real Mean IOP value and a global mean non significantly different from zero (*p*-values > 0.05). Strategy 1 and 5 showed a non significant dependency of the Estimate Error on the Real Mean IOP but had a significant offset (negative for Strategy 1, positive for Strategy 5).

**Table 3:** Estimated regression coefficients of the dependent variable (Estimate Error) on the real Mean IOP value

	*Estimate Error:*				
	Strategy 1	Strategy 2	Strategy 3	Strategy 4	Strategy 5
Mean IOP Coefficient	0.036	−0.226***	0.034	−0.095*	−0.082
	(0.052)	(0.076)	(0.049)	(0.052)	(0.050)
Global Mean	−0.412**	0.978***	0.288*	0.281	0.593***
	(0.175)	(0.255)	(0.166)	(0.175)	(0.168)
Observations	70	70	70	70	70
R^2^	0.007	0.116	0.007	0.047	0.038
Adjusted R^2^	−0.007	0.103	−0.008	0.033	0.024
Residual Std. Error (df = 68)	1.467	2.134	1.389	1.465	1.406
F Statistic (df = 1; 68)	0.487	8.925***	0.475	3.376*	2.708

In brackets, the standard errors of the coefficient estimates. Asterisks represent the significance level according to the legend in the footnotes

*Note:* *p < 0.1; **p < 0.05; ***p < 0.01

#### Fluctuation estimate

As for the Peak Value, Strategies from 2 to 5 were analyzed using simple regression with Gaussian error distribution using the real value as a predictor of the estimate error. The results are reported in Table 4.

**Table 4:** Estimated regression coefficients of the dependent variable (Estimate Error) on the real IOP Fluctuation value

	Estimate Error			
	(2)	(3)	(4)	(5)
IOP Fluctuation Coefficient	−1.010***	−0.720***	−0.759***	−0.759***
	(0.132)	(0.097)	(0.124)	(0.124)
Global Mean	−2.100***	−2.714***	−0.200	−0.200
	(0.383)	(0.281)	(0.360)	(0.360)
Observations	70	70	70	70
R^2^	0.462	0.448	0.356	0.356
Adjusted R^2^	0.454	0.440	0.346	0.346
Residual Std. Error (df = 68)	3.206	2.354	3.008	3.008
F Statistic (df = 1; 68)	58.480***	55.171***	37.562***	37.562***

In brackets, the standard errors of the coefficient estimates. Asterisks represent the significance level according to the legend in the footnotes

*Note:* *p < 0.1; **p < 0.05; ***p < 0.01

**Fig. 3:**
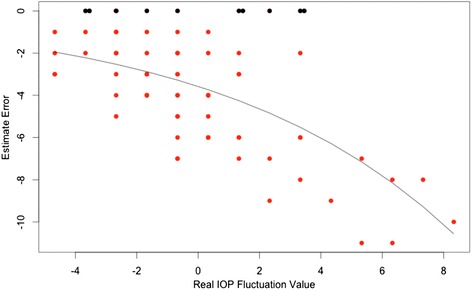
Scatterplot for Estimate Error of Strategy 1. *Black dots* represent the correct estimates (zero error), red dots the incorrect estimates. The *black line* represents the prediction from the ZICP model showing the non linear dependency of the non zero error on the real peak value

As in the Peak estimate, one of the Strategies, specifically Strategy 1, showed a limiting effect to overestimation, yielding a relatively high rate (although much lower than for Strategy 1 for the Peak value) of correct predictions (zero error) and a negatively skewed error distribution. Again, this was modeled using a ZICP model and the odds of correct prediction did not depend on the real fluctuation value (*p*-value = 0.60) and was significantly different from 1 (odds = 0.13, probability of correct prediction = 0.11, *p*-value < 0.01). The mean prediction for non zero errors was significantly dependent on the real IOP fluctuation value (*p* < 0.001) in a non linear fashion, as depicted in Fig. 3.

### Hit or miss analysis

A second step of the analysis was aimed at the characterization, for each strategy, of the probability of yielding clinically reliable estimates of the quantities of interest. To test this, we chose a rage of clinical tolerance (±2 mmHg for the IOP Peak estimate and the Mean IOP estimate, ± 1 for the IOP Fluctuation estimate). Errors within the tolerance range were considered as “Hit”, errors outside the range were counted as “Miss” (subdivided in overestimates, “Over” and underestimates, “Under”). Then, a multinomial logit model was used for each strategy to model the “hit or miss”. This approach had the advantage of allowing a comparison the different strategies independent of the error distribution. Hits were used as the reference category, so the model summary tables (see below) present the logit coefficients of “Over” versus “Hit” and “Under” versus “Hit”.

#### Peak strategies

**Fig. 4:**
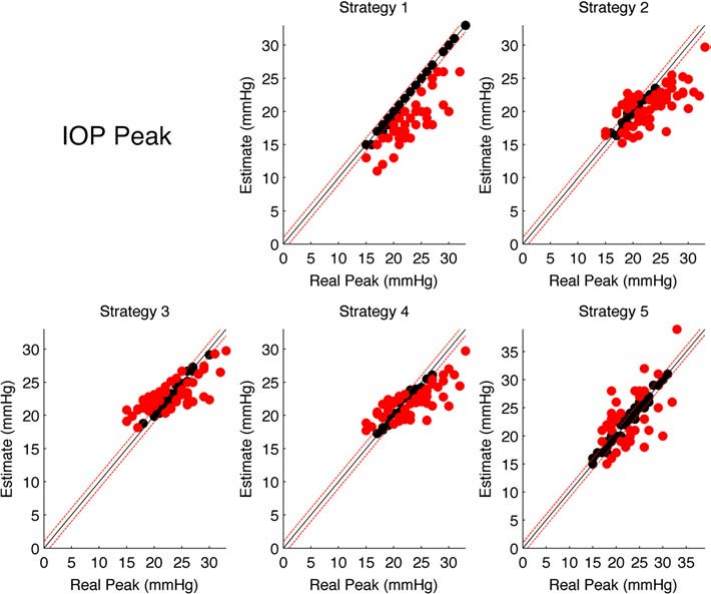
Scatter plots of the estimated IOP Peak for each stratgy versus the real IOP Peak. The *black line* represents the the ideal line of correct predictions, i.e. estimates exactly equal to the real value. *Dashed red lines* represents the range of clinical tolerance (±2 mmHg). *Black dots* represent the “Hits”, while *red dots* represent the “Misses”

Table 5 shows the results for the multinomial logit model for IOP Peak strategies. Each model compares the selected strategy in terms of odds of overestimation or underestimation with respect to correct hits (within ±2 mmHg). The first Strategy had no “Over” since it never overestimated the Peak Value. Strategy 5 resulted to be the most balanced in terms of over and underestimate and showed no significant (*p* > 0.05) relation with the true Peak IOP value (i.e. almost constant error rate) although, as for all the proposed strategies, overestimate tended to cluster at low peak values and underestimate at high peak values. Figure 4 shows scatter plots of the estimated values versus the real values, with misses highlighted in red.

**Table 5:** Estimated logit coefficients from the multinomial logit model (coefficients and standard errors in brackets)

	Over/Underestimate logit				
	Strategy 1	Strategy 2	Strategy 3	Strategy 4	Strategy 5
“Over” at average Peak IOP		−1.395	−0.618	−2.117**	−0.737**
		(0.882)	(0.451)	(0.844)	(0.292)
“Under” at average Peak IOP	0.060	1.486***	−1.441**	0.075	−0.984***
	(0.242)	(0.475)	(0.592)	(0.339)	(0.324)
“Over” True Value Coefficient		−0.295	−0.629***	−0.781***	−0.030
		(0.218)	(0.176)	(0.253)	(0.076)
“Under” True Value Coefficient	0.077	0.611***	0.559***	0.387***	0.122
	(0.061)	(0.171)	(0.180)	(0.123)	(0.076)
Hits	34/70	16/70	21/70	20/70	37/70
Overestimates	0/70	11/70	30/70	19/70	18/70
Underestimates	36/70	43/70	19/70	31/70	15/70

The first two rows report the odds of Overestimates (first row) an Underestimate (second row) with respect to the Hits at the average Peak value. The third and fourth rows report the logit coefficients for Over and Underestimate for the Peak Value (i.e. how the odds vary with the real Peak value). Asterisks represent the significance level according to the legend in the footnotes. The second half of the table reports the actual Hits, Over and Underestimate counts for each strategy

*Note:* *p < 0.1; **p < 0.05; ***p < 0.01

#### Mean strategies

**Fig. 5:**
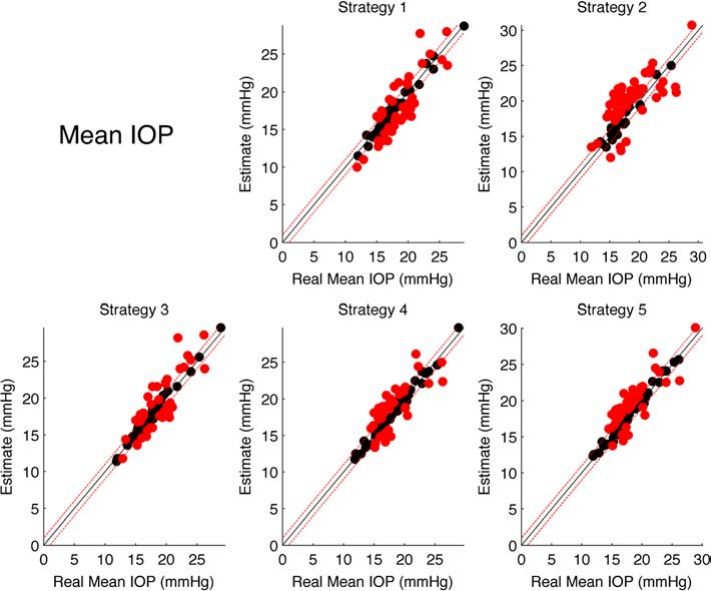
Scatter plots of the estimated Mean IOP for each stratgy versus the real IOP Peak. The *black line* represents the the ideal line of correct predictions, i.e. estimates exactly equal to the real value. *Dashed red lines* represents the range of clinical tolerance (±2 mmHg). *Black dots* represent the “Hits”, while *red dots* represent the “Misses”

Table 6 shows the results for the multinomial logit model for Mean IOP strategies. Each model compares the selected strategy in terms of odds of overestimation or underestimation with respect to correct hits (within ±2 mmHg). Strategy 2 showed the highest rate of Overestimates, being even higher that correct Hits. Strategy 1 showed the highest Hit rate and no correlation of errors with the true Mean IOP value. All strategies except for Strategy 2 showed no significant correlation with the true Mean IOP value. Figure 5 shows scatter plots of the estimated values versus the real values, with misses highlighted in red.

**Table 6:** Estimated logit coefficients from the multinomial logit model (coefficients and standard errors in brackets)

	Over/Underestimate logit				
	Strategy 1	Strategy 2	Strategy 3	Strategy 4	Strategy 5
“Over” at average Mean IOP	−1.141***	0.648**	−0.408	−0.441	−0.167
	(0.346)	(0.285)	(0.279)	(0.282)	(0.258)
“Under” at average Mean IOP	−0.483*	−0.926**	−0.776**	−0.821***	−1.361***
	(0.272)	(0.452)	(0.313)	(0.318)	(0.393)
“Over” True Value Coefficient	0.137	0.080	0.104	−0.096	−0.011
	(0.095)	(0.100)	(0.083)	(0.092)	(0.079)
“Under” True Value Coefficient	0.052	0.355***	0.070	0.063	0.113
	(0.083)	(0.126)	(0.095)	(0.087)	(0.102)
Hits	36/70	21/70	33/70	33/70	33/70
Overestimates	12/70	38/70	22/70	22/70	28/70
Underestimates	22/70	11/70	15/70	15/70	9/70

The first two rows report the odds of Overestimates (first row) an Underestimate (second row) with respect to the Hits at the average Mean IOP value. The third and fourth rows report the logit coefficients for Over and Underestimate for the Mean IOP Value (i.e. how the odds vary with the real Mean IOP value). Asterisks represent the significance level according to the legend in the footnotes. The second half of the table reports the actual Hits, Over and Underestimate counts for each strategy

*Note:* *p < 0.1; **p < 0.05; ***p < 0.01

#### Fluctuation strategies

**Fig. 6:**
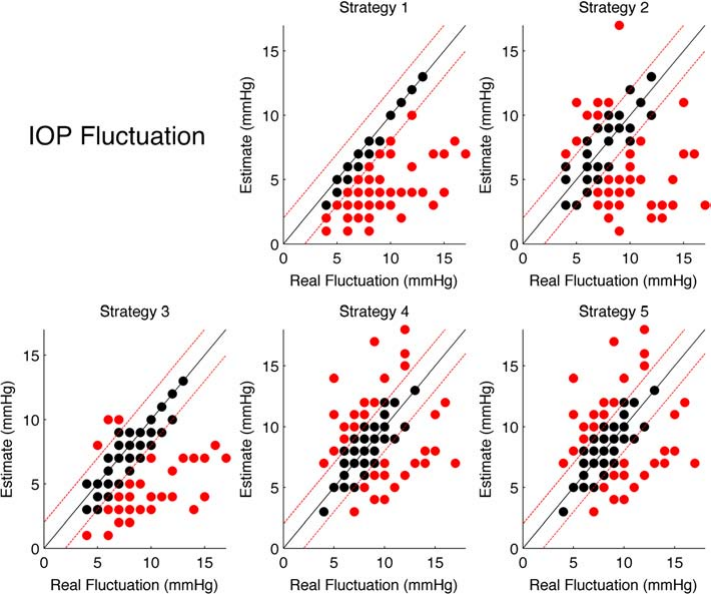
Scatter plots of the estimated IOP Fluctuations for each stratgy versus the real IOP Peak. The *black line* represents the the ideal line of correct predictions, i.e. estimates exactly equal to the real value. *Dashed red lines* represents the range of clinical tolerance (±1 mmHg). *Black dots* represent the “Hits”, while *red dots* represent the “Misses”

Table 7 shows the results for the multinomial logit model for IOP Fluctuation strategies. Each model compares the selected strategy in terms of odds of overestimation or underestimation with respect to correct hits (within ± mmHg). The strategies with the highest Hit rate and more balanced Over/Underestimation rate were Strategy 4 and 5 (which yielded the exact same estimates for all subjects), although showing a strong correlation with the real IOP fluctuations, especially for the odds of underestimations, being more likely for higher Fluctuation value. Figure 6 shows scatter plots of the estimated values versus the real values, with misses highlighted in red.

**Table 7:** Estimated logit coefficients from the multinomial logit model (coefficients and standard errors in brackets)

	Over/Underestimate logit				
	Strategy 1	Strategy 2	Strategy 3	Strategy 4	Strategy 5
“Over” at average IOP Fluctuation		−2.040***	−3.218***	−0.870**	−0.870**
		(0.641)	(1.098)	(0.343)	(0.343)
“Under” at average IOP Fluctuation	0.700**	−0.214	0.089	−1.329***	−1.329***
	(0.277)	(0.296)	(0.258)	(0.426)	(0.426)
“Over” True Value Coefficient		−0.438*	−0.460	−0.109	−0.109
		(0.235)	(0.356)	(0.147)	(0.147)
“Under” True Value Coefficient	0.294**	0.470***	0.248**	0.634***	0.634***
	(0.115)	(0.141)	(0.102)	(0.174)	(0.174)
Hits	25/70	32/70	32/70	36/70	36/70
Overestimates	0/70	9/70	3/70	17/70	17/70
Underestimates	45/70	29/70	35/70	17/70	17/70

The first two rows report the odds of Overestimates (first row) an Underestimate (second row) with respect to the Hits at the average Mean IOP value. The third and fourth rows report the logit coefficients for Over and Underestimate for the Mean IOP Fluctuation value (i.e. how the odds vary with the real IOP Fluctuation value). Asterisks represent the significance level according to the legend in the footnotes. The second half of the table reports the actual Hits, Over and Underestimate counts for each strategy

*Note:* *p < 0.1; **p < 0.05; ***p < 0.01

### Treatment group analysis

In general, no significant differences were found among the treatment groups. The only exceptions were for Strategy 1 for the Peak IOP, where a significant difference in the Estimate Error was found between the Brimonidine and the Latanoprost group (Latanoprost Mean Error = −3.01, Brimonidine Mean Error = −1.4, *p* = 0.0105), for Strategy 1 for the IOP Fluctuations, where a significant difference in the Estimate Error was found between the Timolol and the Latanoprost group (Latanoprost Mean Error = −4.41, Timolol Mean Error = −3.37, *p* = 0.0045) and for Strategy 3 for the IOP Fluctuations, where a significant difference in the Estimate Error was found between the Cosopt and the Latanoprost group (Latanoprost Mean Error = −1.52, Cosopt Mean Error = −4.33, *p* = 0.0095). A logit regression model was also calculated for each Strategy to test differences in Hit/Miss rate among the treatment groups, but no significant difference among treatment groups could be detected for all strategies.

### Hit rate comparison of the strategies

For each considered feature (Peak, Mean and Fluctuations) all strategies were compared in terms of Hits using a logit model corrected for repeated measures with a Subject random effect and a post hoc analysis. For the Peak strategies Strategy 5 yielded the highest Hit count (37/70) and resulted to be significantly different from Strategies 2, 3 and 4 (corrected *p* < 0.05). Strategy 1 resulted to be significantly different from Strategy 2 (*p* = 0.0079) with a higher Hit count, but not from Strategy 3, 4 and 5 (although the comparisons with strategies 3 and 4 had low *p*-values, respectively 0.11 and 0.07). For the Mean IOP, only Strategy 1 resulted to be significantly different from Strategy 2 (*p* = 0.031) but any other difference was not significant, with Strategy 1 being the one with the highest Hit count (36/70). No significant differences could be found between the Strategies for the IOP Fluctuations, Strategy 4 and 5 being the ones with the highest Hit count (36/70).

## Discussion

The results of this study on glaucoma patients treated with different IOP lowering eye drops remark the concept that IOP is a dynamic parameter and that intensive measurement is helpful in determining its characteristics.

Our dataset confirms the critical role of IOP variations during night hours in glaucoma: IOP peaked outside office-hour in 65 %, which is similar to 52–66 % of other studies [13, 19, 25]. This remarks the importance of considering the 24-hour characteristics of IOP, at least in critical patients, in order to better tailor treatments to individual IOP patterns [15].

Different strategies to predict the 24-hour rhythm of IOP have been reported in literature [14, 16, 18, 19]. In general, supine IOP is higher than sitting IOP due to increased episcleral venous pressure, and the concept that supine measurement may be used to predict 24-hour IOP peak dates 1975 [26].

Correction formulas to predict peak from both sitting [16] and supine [14, 16] measurements have also been suggested. Water drinking test [27] and ibopamine [28] have also been suggested to predict peak IOP. The strategy used in this study (a combination of office-hour supine and sitting measurements) had been previously used on untreated subjects [14], and we clearly showed that the relevant 24-hour IOP characteristics that can be missed by routine examination (ie office-hour sitting measurements) can be frequently detected when sitting and supine readings are associated.

When compared to our previous report, this study seems to suggest that, in general, office hour measurements are not adequate to correctly estimate peak, mean and fluctuations values within the acceptance thresholds considered in this paper in treated glaucoma patients.

The best office-hour strategy to estimate the peak IOP was the combination of sitting and supine office hour measurements (strategy 5). The error in this strategy was the least correlated with the real IOP value when compared with other strategies. In addition it was not affected by a significant mean offset (i.e. yelding the overall least biased estimate).

Strategy 1 and 5 were similar in terms of number of correct peak predictions and resulted not to be significantly different upon the correct prediction analysis. It is important to notice, although, that strategy 1 was greatly affected by the highly negatively skewed distribution (i.e. allowed only underestimations) which were not counterbalanced by the zero error predictions (34 %). From a clinical point of view this is of particular importance, showing that sitting office hour measurements tend to underestimate the correct peak value. This error is not only significantly correlated with the real Peak IOP value, but its non linear relation with the real Peak IOP is difficult to model and to compensate for.

All office-hour strategies for the Mean IOP estimate, except Strategy 2, showed similar features. The best strategy in terms of number of correct predictions was sitting office hour measurments alone (strategy 1), although the odds of correct predictions were not significantly different from strategies 3, 4 and 5. Strategies 1, 3 and 4 could be considered equal since no correlation of the errors with the real values or significant mean offset (i.e. no systematic bias) could be found; they performed equally in terms of correct predictions (about 50 %) and the wrong prediction rate was not correlated with the real Mean IOP values. Strategy 5 had a significant mean offset (0.6 mmHg) but this did not affect the correct prediction rate significantly. Strategy 2 was the worst both in terms of correct predictions and in terms of significant systematic bias (especially, exhibited a strong correlation of the errors with the real Mean IOP value).

The IOP Fluctuation strategies were affected by several flaws. Strategy 1 performed very poorly in terms of correct predictions and, as for Strategy 1 for the Peak IOP, suffered from a non linear strong correlation of the error rate with the real IOP value and a skewed error distribution, allowing only underestimates. The best in terms of correct predictions were strategies 4 and 5 (which yielded the same estimates for all subjects) although they were not significantly different from the other strategies. All strategies showed a strong correlation of the error with the real IOP Fluctuation value, underestimating higher values.

As shown in Table 8 using sitting office-hour measurements, a correct identification of all parameters (peak, mean and fluctuation) was achieved in 24 % of cases, which was very similar to untreated patients (20 %) [14], with a slight increased percentage of cases fully characterized adding supine office-hour values (27 %).

**Table 8:** The Clinical Impact: Improvement in the Characterization of the 24-Hour Curve Using Different Criteria

	All patients
Fully characterized by sitting office-hour estimates	24
Fully characterized by sitting + supine office-hour estimates	27
Fully characterized by sitting office-hour estimates + peak estimation	22
Supine + sitting office hour strategy at least partially improves office-hour sitting estimates	66
Supine office-hour estimates + peak estimation strategy* at least partially improves office-hour sitting estimates	30
Fully uncharacterized	9

Data are percentages

Full characterization: mean IOP, peak, and fluctuations, respectively, within 1, 1, 2 mm Hg from the 24-hour value

Full absence of characterization: mean IOP, peak, and fluctuations, respectively, outside 1, 1, 2 mm Hg from the

24-hour value

The analyses of this study may be prone to a number of possible limitations that have been previously described and discussed in details [14]^.^ The accuracy of 24-hour data may be affected by a number of factors, including hospitalization, disturbed sleep, sudden waking and exposure to light at night [29, 30]. The use of two tonometers could also be a specific limit of this paper [31, 32]. Still, this 24-hour procedure is strictly standardized and controlled, and it has been largely used in our center for nearly two decades [14, 24, 33–36]. Different characteristics of the study groups (in particular, baseline IOP) may largely influence our findings. In fact, we found that mean 24-hour IOP was higher with timolol than with others glaucoma medications, thus confirming previous findings, showing a substantial effect of timolol during the day, but no measurable effect at night [24]. Also the choice of timepoints was critical in determining the results: in particular, latanoprost patients received their medication after the 9-pm-measurement, a fact that could explain the high percentage of IOP peaks outside office-hours. Adherence to treatment was not a concern, as medications were administered by study personnel during the study period.

## Conclusions

The results of this study remark the concept that IOP is a dynamic parameter and that intensive measurement is helpful in determining its characteristics. The methods of this study have been previously used for untreated subjects, for whom we clearly showed that relevant 24-hour IOP characteristics may be missed by routine examination (ie office-hour sitting measurements), whereas a combination of office-hour sitting and supine measurements can provide more accurate information.

The results of this study on treated glaucoma patients show a very poor performance of all office-hour strategies in correctly predicting the considered parameters within the thresholds used in this paper, all scoring a correct prediction rate below 52 %. Some strategies allowed a simple linear modeling. In these cases a model to correct for the systematic bias could be theoretically applicable, but a much larger sample size would be required for precise parameter estimation. In all cases, however, the residual standard error was relatively high (ranging from 1.4 to 3.2) so that a model correction (i.e. zero mean error) would hardly succeed in obtaining reliable estimates.

## Abbreviations

IOP: intraocular pressure
POAG: primary open angle glaucoma
FCDT: fixed combination dorzolamide timolol
